# Collagen/β_1_ integrin interaction is required for embryoid body formation during cardiogenesis from murine induced pluripotent stem cells

**DOI:** 10.1186/1471-2121-14-5

**Published:** 2013-01-25

**Authors:** Di Zeng, Dong-Bo Ou, Ting Wei, Lu Ding, Xiong-Tao Liu, Xin-Lin Hu, Xue Li, Qiang-Sun Zheng

**Affiliations:** 1 Department of Cardiology, Tangdu Hospital, Fourth Military Medical University, Xi'an, 710038, China

**Keywords:** Collagen/β_1_ integrin interaction, Embryoid body, Cardiac differentiation, Induced pluripotent stem cell

## Abstract

**Background:**

The interactions between stem cells and extracellular matrix (ECM) mediated by integrins play important roles in the processes that determine stem cell fate. However, the role of ECM/integrin interaction in the formation of embryoid bodies (EBs) during cardiogenesis from murine induced pluripotent stem cells (miPSCs) remains unclear.

**Results:**

In the present study, collagen type I and β_1_ integrin were expressed and upregulated synergistically during the formation of miPSC-derived EBs, with a peak expression at day 3 of differentiation. The blockage of collagen/β_1_ integrin interaction by β_1_ integrin blocking antibody resulted in the production of defective EBs that were characterized by decreased size and the absence of a shell-like layer composed of primitive endoderm cells. The quantification of spontaneous beating activity, cardiac-specific gene expression and cardiac troponin T (cTnT) immunostaining showed that the cardiac differentiation of these defective miPSC-derived EBs was lower than that of control EBs.

**Conclusions:**

These findings indicate that collagen/β_1_ integrin interaction is required for the growth and cardiac differentiation of miPSC-derived EBs and will be helpful in future engineering of the matrix microenvironment within EBs to efficiently direct the cardiac fate of pluripotent stem cells to promote cardiovascular regeneration.

## Background

Embryonic stem cells (ESCs) are characterized by unlimited proliferation capacity and multipotency and are one of the most promising stem cell populations for use in cardiovascular regenerative medicine. However, the use of ESCs is hampered by their embryonic origin and by the potential immune rejection of allogeneic cell grafts. These issues may potentially be circumvented by the use of induced pluripotent stem cells (iPSCs) that are reprogrammed from somatic cells by the ectopic expression of 4 transcription factors (Oct4, Sox2, c-Myc, and Klf4) [1,2]. The use of iPSCs allows the generation of patient-specific cells of any lineage that is not derived from embryos. iPSCs have a differentiation capacity similar to that of ESCs and were shown to be capable of differentiating into functional cardiomyocytes through embryoid body (EB) formation [3,4]. EB formation has been utilized widely as a trigger of in vitro differentiation in ESCs and iPSCs and is a critical intermediate step in the induction of lineage-specific differentiation [5]. The importance of EB formation is further demonstrated by findings that lineage-specific differentiation programs within EBs closely resemble lineage commitment programs in the developing embryo [6,7].

Lineage-specific differentiation within EBs is a response to a variety of environmental stimuli, including cell-cell adhesion and cell-matrix interactions. Recent strategies for engineering EB formation have focused primarily on controlling EB size, extracellular matrix (ECM) interactions and cell-cell adhesion [8]. Processes that determine cell fate are often mediated by the ECM, and such mediation has been studied largely by seeding ESCs or EBs within natural ECM hydrogel materials [9,10]. Native ECM components such as collagen, fibronectin and laminin direct cell differentiation through integrin-mediated signaling events [11] and through intracellular signaling cascades that ultimately lead to gene expression changes that modulate cell phenotype [12]. ECM/integrin interactions have been shown to influence stem cell differentiation, attachment and proliferation during early embryonic development [9,13]. However, little is known regarding the role of ECM/integrin interactions in the growth and cardiac lineage commitment of iPSC-derived EBs that closely resemble corresponding processes in the developing embryo. The objective of the present study was to explore the role of collagen/β_1_ integrin interaction in EB formation from miPSCs and the involvement of such interaction in the differentiation of miPSCs into cardiomyocytes.

## Results

### Collagen and β_1_ integrin are expressed synergistically during EB formation

**Figure 1:**
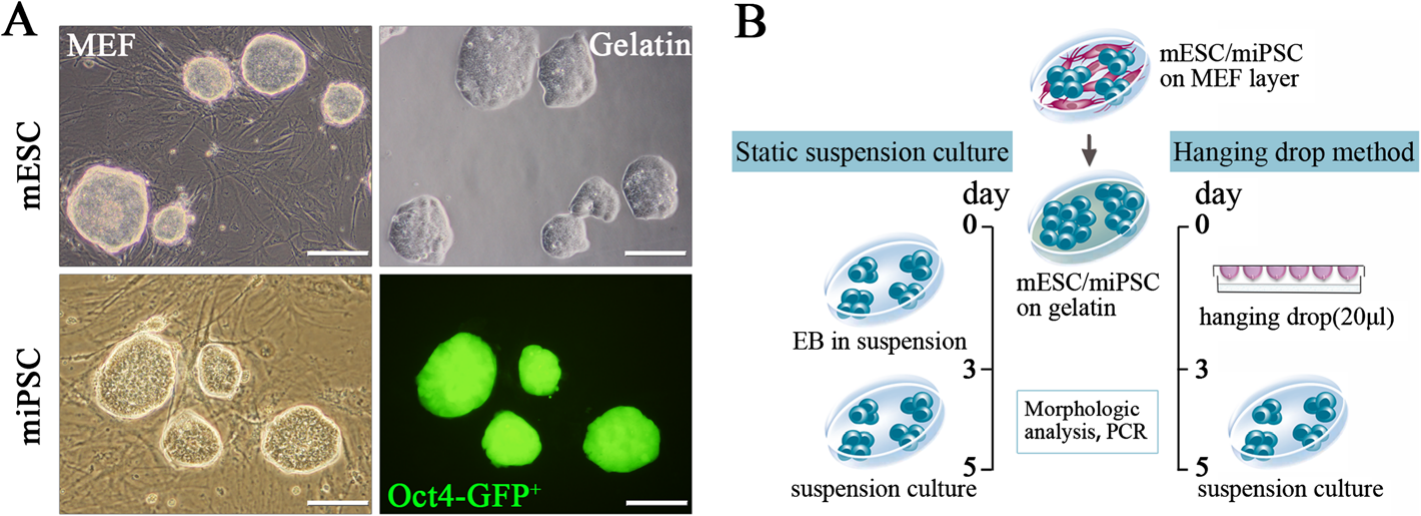
**Cell culture of mESCs and miPSCs. (A)** Morphology of undifferentiated miPSCs and mESCs cultured on MEF feeder layers or gelatin-coated plates. **(B)** Scheme of EB formation via the hanging drop method and static suspension culture as described in Materials and Methods .

**Figure 2:**
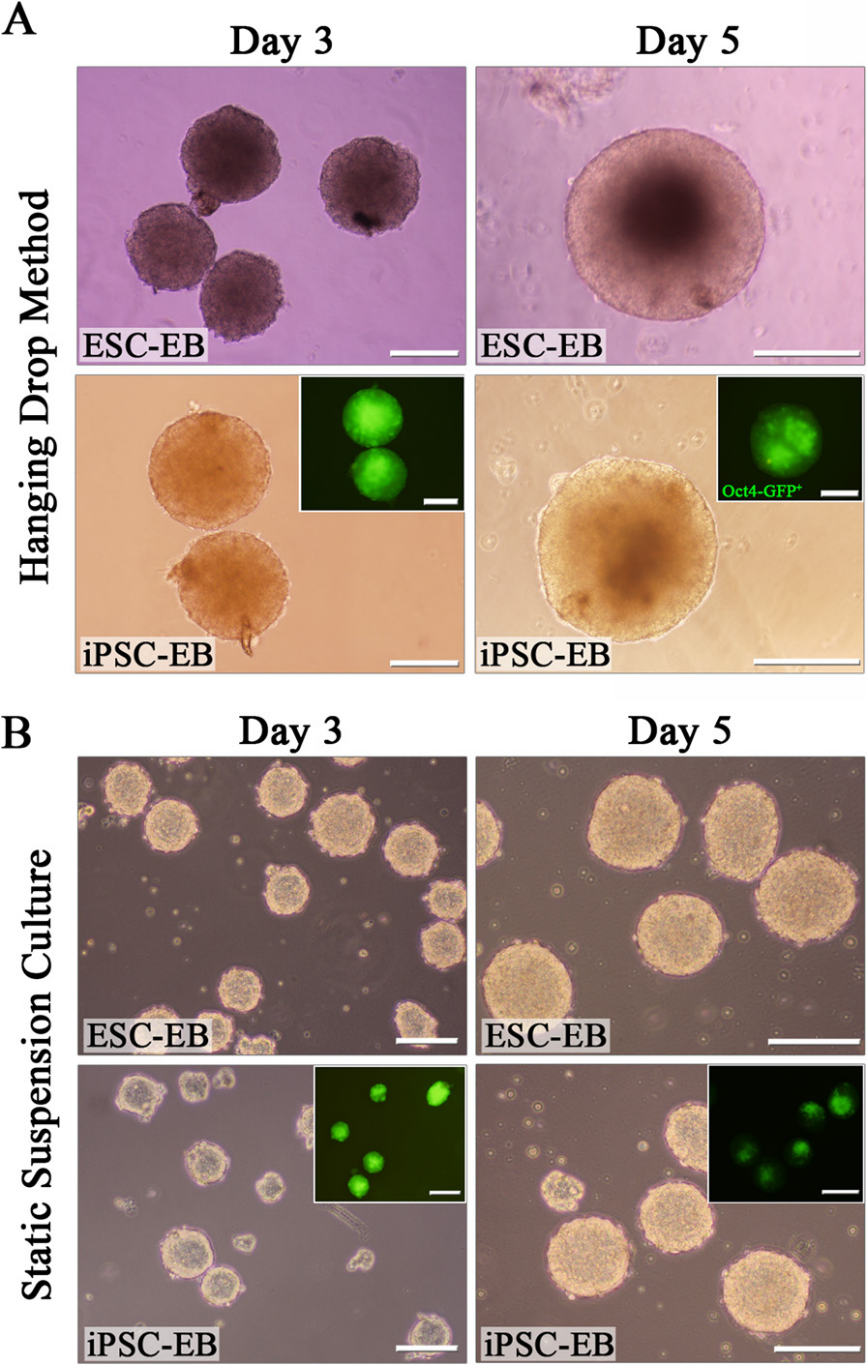
**EB formation by mESCs and miPSCs.** 3-day and 5-day-old EBs derived from mESCs and miPSCs via the hanging drop method **(A)** and static suspension culture **(B)**. Insets: fluorescent micrographs of miPSC-derived EBs that expressed Oct4-eGFP. Scale bars = 200 μm.

The morphology of the undifferentiated miPSC colonies carrying the GFP transgene targeted to the Oct4 locus closely resembled that of the mESCs growing on either MEFs or gelatin-coated dishes under feeder-free conditions and expressing green fluorescent protein (GFP) (Figure 1A). To induce cardiac differentiation, the miPSCs and mESCs were cultured via the hanging drop method or static suspension to form three-dimensional embryoid bodies (EBs) (Figure 1B). Through both of these culture systems, EBs resembling those derived from mESCs were generated from the miPSCs on day 3 and 5 of differentiation (Figure 2A, B).

**Figure 3:**
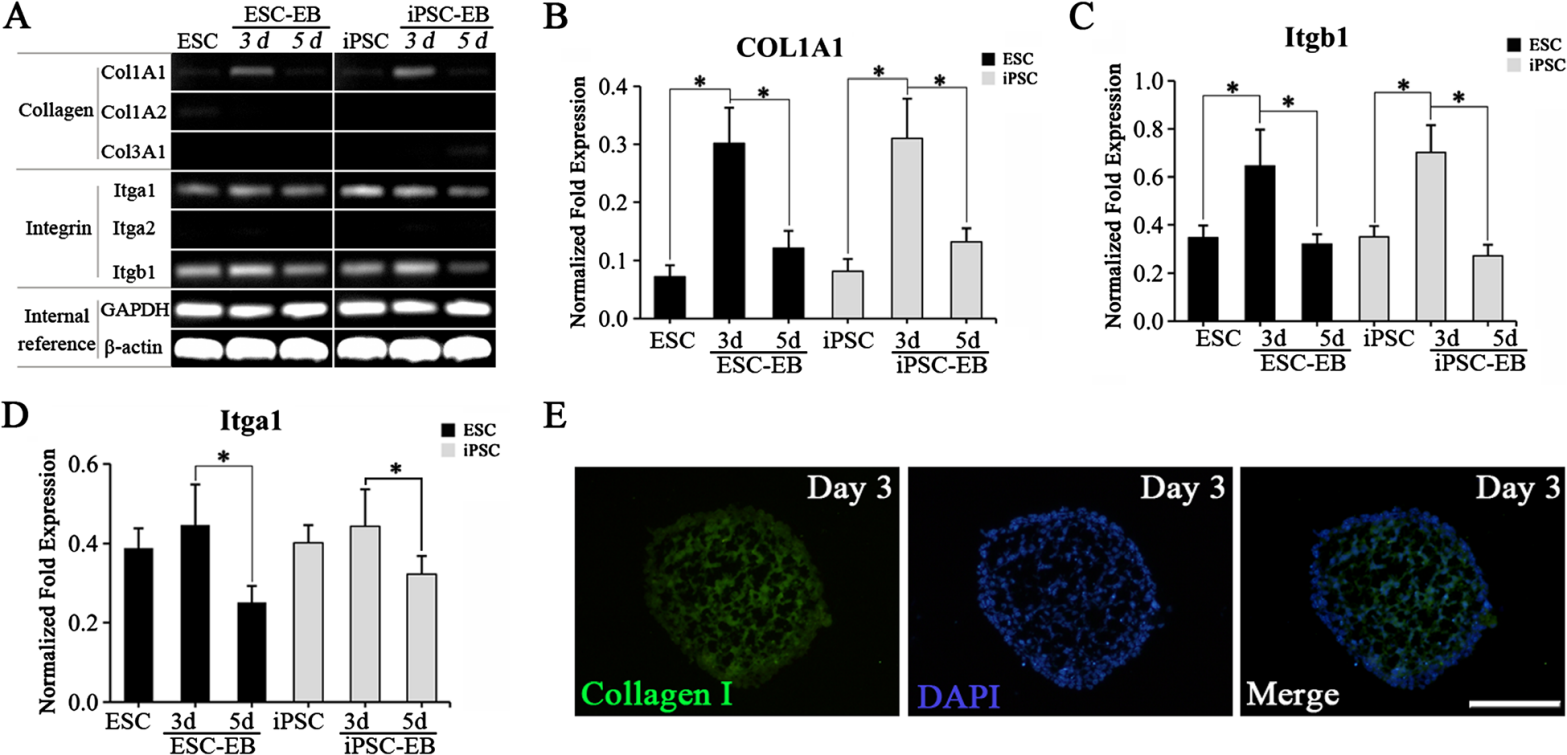
**Expression patterns of collagen and integrins during EB formation. (A)** mRNA expression of collagen (Col1A1, Col1A2, Col3A1) and its integrin receptors (Itga1, Itga2, Itgb1) determined by semiquantitative RT-PCR. **(B to D)** Real-time PCR analysis of Col1A1, Itga1 and Itgb1, normalized to β-actin, in mESCs, miPSCs, and EBs (days 3 and 5). Experiments were performed in triplicate. *Significant at *p* < 0.05. **(E)** Immunostaining of collagen type I in miPSC-derived EBs on day 3. Scale bars = 200 μm.

To explore the role of collagen/integrin interactions in EB formation, the expression of collagen and of its integrin receptors (α_1_, α_2_, β_1_) was determined by semiquantitative RT-PCR in undifferentiated mESCs, miPSCs, and EBs. Undifferentiated mESCs and miPSCs expressed Itga1 and Itgb1 (integrin α_1_, β_1_), whereas Col1A1, Col1A2, Col3A1 and Itga2 were not expressed (Figure 3A). During EB formation, the expression of Col1A1 and Itgb1 was upregulated and peaked on day 3 of differentiation, then fell to levels equal to those in mESCs and miPSCs on day 5 (Figure 3A). Real-time PCR results confirmed that Col1A1 and Itgb1 were expressed in a temporal pattern with a peak on day 3 during EB formation (Figure 3B, C). The expression of Itga1 (an α subunit of collagen receptor) was not upregulated but reduced on day 5 during EB formation (Figure 3D). Immunostaining of EBs on day 3 demonstrated the expression of collagen type I (Figure 3E). These findings suggest that collagen type I and β_1_ integrin were expressed temporarily and synergistically during EB formation in mESCs and miPSCs.

### β_1_ integrin blocking inhibits the growth and shell formation of miPSC-derived EBs

**Figure 4:**
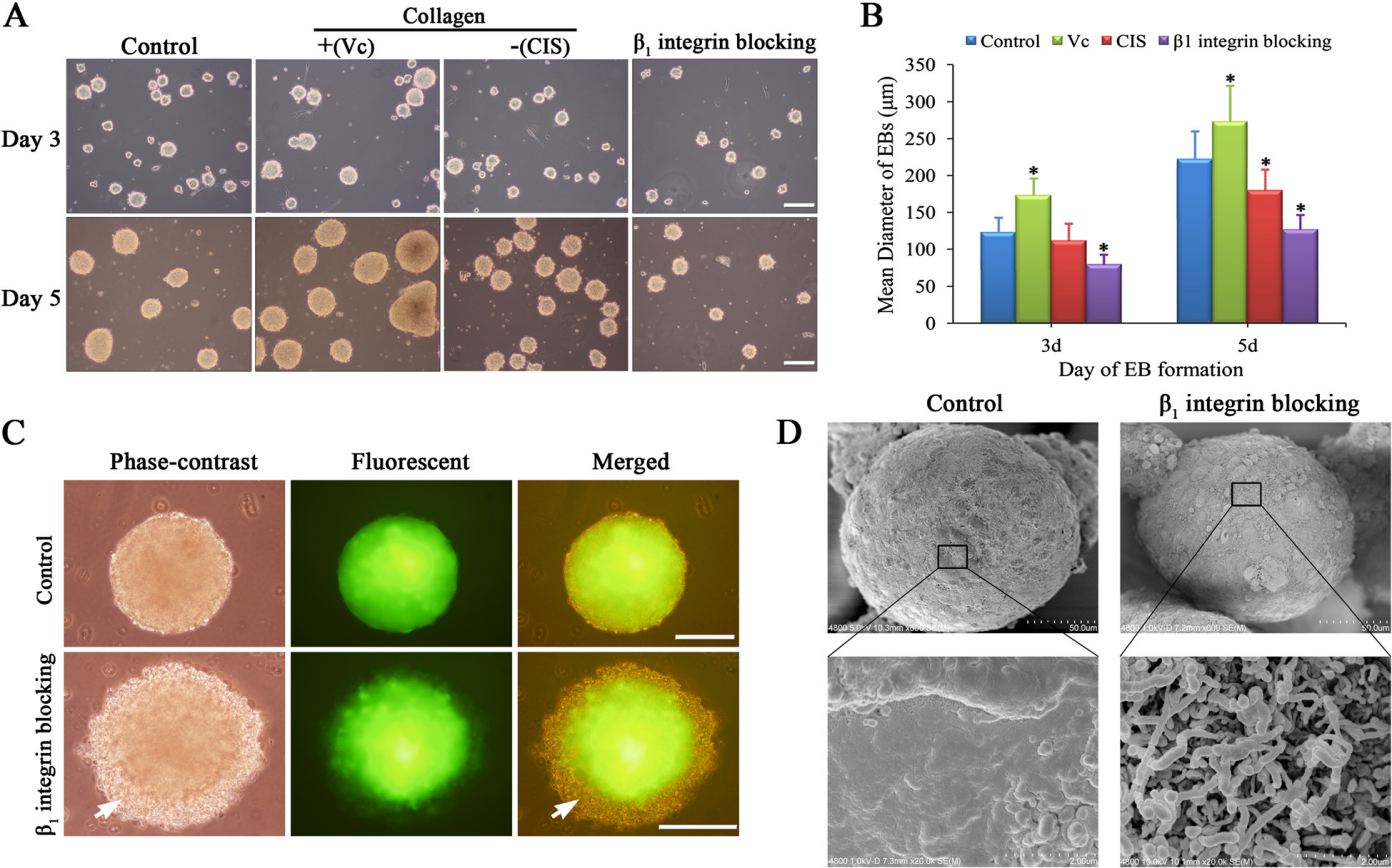
**Blocking of collagen/β**_**1**_**integrin interaction inhibits the growth and shell formation of miPSC-derived EBs. (A)** Morphology of miPSC-derived EBs after treatment with Vc (collagen stimulator), CIS (collagen inhibitor) or β_1_ integrin blocking antibody. β_1_ integrin blocking resulted in delayed growth of EBs and a rough edge. **(B)** Size analysis revealed that the mean diameter of EBs, compared with that of the control group, was larger in the Vc group and smaller in the CIS and β_1_ integrin blocking groups (*p < 0.05). The data were obtained from five sample EBs per field in 10 fields, and three independent experiments were performed. **(C)** Phase-contrast and fluorescent micrographs of miPSC-derived EBs grown as hanging drop with or without β_1_ integrin blocking. Arrows indicate the loose outer layer and the absence of a dense shell. **(D)** Scanning electron micrographs of 5-day-old EBs with or without β_1_ integrin blocking. A fibrous ECM coating and a smooth exterior were observed in control EBs (left column). In contrast, the surface topography of EBs with β_1_ integrin blocking was characterized by a cilium-like structure and the absence of a dense shell-like layer (right column). Scale bars = 200 μm.

Because ECM/integrin interactions have been shown to influence stem cell differentiation during early embryonic development [9,13], we hypothesized that collagen/β_1_ integrin interaction may play a similar role in EB formation, which closely resembles embryonic development. To test this hypothesis, we administered 100 μM Vc, 300 μM CIS and 10 ng/ml β_1_ integrin blocking antibody to respectively promote collagen synthesis, inhibit collagen synthesis, and block collagen/β_1_ integrin interaction prior to EB formation (Figure 4A). Size analysis showed that the mean diameter of the EBs produced in static suspension culture was larger in the Vc group and smaller in the CIS and β_1_ integrin blocking groups compared with the control group (*P* < 0.05, Figure 4A, B). On day 5 of differentiation, the mean diameter of EBs was 263.3 ± 59.6 μm in the Vc group, 179.6 ± 30.3 μm in the CIS group, and 222.7 ± 42.1 μm in the control group. β_1_ integrin blocking led to a marked decrease in EB size to 127.0 ± 22.2 μm (Figure 4B). The mean diameter of EBs produced via the hanging drop method was not significantly altered by β_1_ integrin blocking (data not shown). Phase-contract and fluorescent micrographs of miPSC-derived EBs formed after β_1_ integrin blocking revealed a loose outer layer lacking a dense shell as observed in control EBs (Figure 4C). Scanning electron micrographs revealed the formation of a smooth outer layer resulting from extracellular matrix deposition in control EBs (day 5), in contrast to a cilium-like surface topography lacking a dense shell-like layer in EBs formed after β_1_ integrin blocking (Figure 4D).

### Decreased cardiac differentiation of miPSC-derived EBs after β_1_ integrin blocking

**Figure 5:**
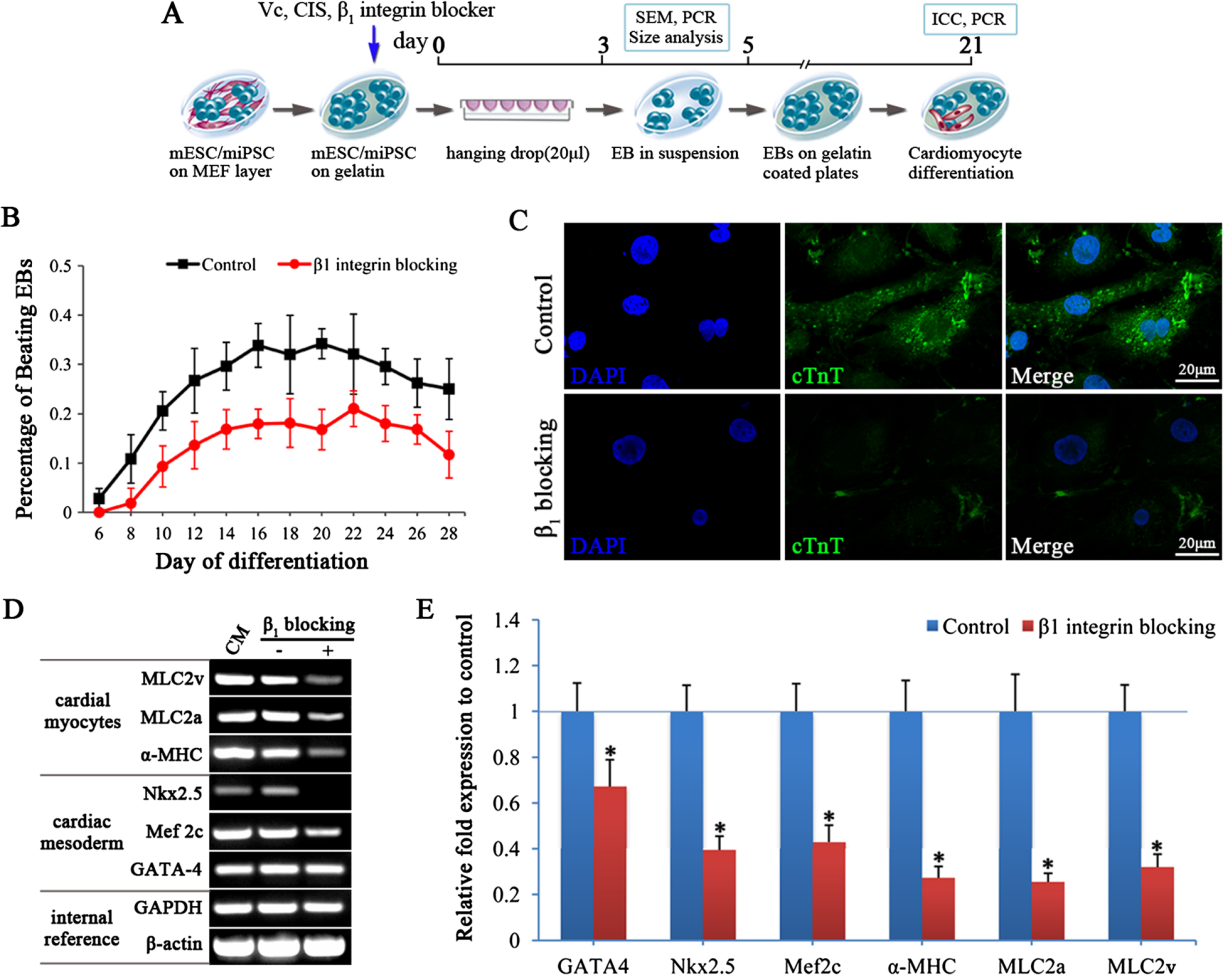
**Decreased cardiac differentiation of miPSC-derived EBs formed after blocking of collagen/β**_**1**_**integrin interaction. (A)** Schematic protocol of cardiac differentiation from miPSCs. Vc, CIS or β_1_ integrin blocking antibody was administered prior to EB formation. **(B)** Time course quantification of spontaneous beating activity of differentiated cardiomyocytes was expressed as the percentage of beating EBs. Error bars indicate SEM. **(C)** Immunostaining for cardiac troponin-T (cTnT) of miPSC-derived cardiomyocytes on day 21. Nuclei were counterstained with DAPI (blue). Scale bars = 20 μm. **(D, E)** Gene expression profiles of cardiac-related transcription factors (GATA-4, Mef2c, Nkx2.5) and cardiac-specific structural genes (α-MHC, MLC2a, MLC2v) were analyzed by semiquantitative RT-PCR (D) and real-time PCR (E). Treatment with β_1_ integrin blocking antibody decreased the expression of these factors and genes. Expression levels of each gene were normalized to β-actin or GAPDH. Mean fold change relative to control and SD from triplicate experiments are shown. *p < 0.05 relative to control.

To assess whether blocking of collagen/β_1_ integrin interaction during EB formation affects the cardiac lineage commitment of miPSCs, miPSC-derived EBs formed with or without β_1_ integrin blocking were differentiated using a standard protocol (Figure 5A). From day 6 to day 28 of differentiation, plated EBs were examined for spontaneous beating activity. During the time course of cardiac differentiation, the percentage of beating EBs in the β_1_ integrin blocking group was lower than that in the control group (Figure 5B). The percentage of beating EBs continued to increase until day 16 of differentiation and reached plateau values of 18.0 ± 3.0% in the β_1_ integrin blocking group and 33.8 ± 4.4% in the control group. On day 21, the beating EBs were immunostained for cardiac troponin T (cTnT) to characterize the cardiac-specific protein. This immunostaining revealed organized sarcomeric myofilaments in the control group in contrast to less cTnT staining and unorganized myofilaments in the β_1_ integrin blocking group (Figure 5C).

To characterize the cardiomyocyte phenotype, we included a series of marker genes in the study. GATA-binding protein 4 (GATA-4), NK2 transcription factor related locus 5 (Nkx2.5), and myocyte enhancer factor 2C (Mef2c) were included as markers for cardiac mesoderm. α-myosin heavy chain (α-MHC) and myosin light chain 2 atrial and ventricular transcripts (MLC2a, MLC2v) were included as markers for cardiomyocytes. The semiquantitative RT-PCR and real-time PCR results both demonstrated that all of the above cardiac-specific markers were decreased significantly in the miPSC-derived EBs formed after β_1_ integrin blocking (Figure 5D, E). These findings indicate that blocking of collagen/β_1_ integrin interaction during EB formation leads to decreased cardiac differentiation of miPSCs.

## Discussion

EB formation, which recapitulates various aspects of early embryonic development and displays a high degree of self-organization [14], has been commonly used to induce lineage-specific differentiation of ESCs and iPSCs. The typical characteristics of EB formation, such as EB size and shape, are important parameters that influence cardiac lineage commitment [15-17]. Collagen, the major component of ECM, has been shown to enhance cardiac cell engraftment [18] and cardiac differentiation [19] through integrin-mediated interactions between the cells and collagen. There is increasing evidence that ECM/integrin interactions are essential for stem cell differentiation, attachment and proliferation during early embryonic development [9,13]. We hypothesized that collagen/β_1_ integrin interaction plays an essential role in the formation and cardiac differentiation of iPSC-derived EBs, which closely resembles early embryonic development. To test this hypothesis, we examined the expression patterns of collagen and integrins during EB formation. Our findings, consistent with those of a previous study [20], showed that undifferentiated mESCs and miPSCs expressed integrin α_1_ and β_1_ but not integrin α_2_. During EB formation, collagen and β_1_ integrin were upregulated synergistically and peaked on day 3 of differentiation, suggesting that the collagen/β_1_ integrin interaction may be involved in EB formation.

We used two common culture techniques, the hanging drop method and static suspension. Static suspension culture is performed by adding a suspension of ESCs or iPSCs to a bacteriological Petri dish and allowing the cells to aggregate spontaneously via cell-cell adhesion [21]. This method was used to assess the effect of collagen/β_1_ integrin interaction on the growth of miPSC-derived EBs. The size and shape of the EBs resulting from static suspension culture tend to be heterogeneous [21], and this phenomenon has been shown to influence cardiac lineage-specific differentiation [15-17]. Such heterogeneity is avoided by the hanging drop method, in which a defined number of isolated mESCs or miPSCs are added to a drop where they aggregate [22]. To exclude the confounding effect of size heterogeneity on cardiac differentiation, we used the hanging drop method to investigate the effect of collagen/β_1_ integrin interaction on the cardiac differentiation of miPSCs.

The size analysis of EBs formed by static suspension culture showed that EB size was increased by treatment with ascorbic acid (a collagen stimulator) but decreased by treatment with CIS (a collagen inhibitor), indicating that collagen synthesis is potent regulator of EB growth. Treatment with β_1_ integrin blocking antibody produced a greater decrease in EB size, indicating the essential role of collagen/β_1_ integrin interaction in the control of EB size. The size of EBs formed by the hanging drop method was not affected by treatment with β_1_ integrin blocking antibody. However, the EBs formed by this method following β_1_ integrin blocking did not form a dense shell-like layer as is observed in normal EBs [23,24]. During EB formation, a layer of primitive endoderm is often formed on the exterior surface following cell aggregation [25]. Consistent with our observation that EBs failed to form a dense shell-like layer following β_1_ integrin blocking, another recent study demonstrated that β_1_ integrin blocking in differentiating EBs resulted in the detachment of this endoderm layer from the EB surface [26].

Because EB size and shape have been shown to play essential roles in cardiac lineage commitment [15-17], we investigated whether β_1_ integrin blocking during EB formation affects the cardiac lineage commitment of miPSCs. A study of spontaneous beating activity, the quantification of cardiac-specific gene expression and cTnT immunostaining all revealed decreased cardiac differentiation in miPSC-derived EBs formed after the blocking of collagen/β_1_ integrin interaction. In addition, we also assessed some markers related to the structural/maturity characteristics of cardiomyocytes, such as cTnI, MLC2a, and MLC2v. cTnT staining showed that iPSCs-derived cardiomyocytes failed to form organized sarcomeric myofilaments with β_1_ integrin blocking as that in the control group (Figure 5C), and measurement of MLC2a and MLC2c revealed decreased expression after integrin β_1_ disruption (Figure 5D, E). Moreover, organized myofilaments and high MLC2v expression are also considered as markers for mature cardiomyocytes [27]. These results suggested that the structure and maturity of iPSCs-derived cardiomyocytes were impaired by integrin β_1_ blocking. In conclusion, our findings indicate that collagen/β_1_ integrin interaction is required for the growth and cardiac differentiation of miPSC-derived EBs. This information will be helpful in future engineering of the EB microenvironment to promote the cardiac differentiation of pluripotent stem cells.

In the developing embryo, the native ECM plays a central role by mediating biophysical stimuli, biochemical and molecular signals and spatial organization. The process of constant interchange between cells and the ECM, termed dynamic reciprocity, determines cell fate and triggers the shift from proliferation to structure formation [28]. As main components of native ECM, collagen may be involved in the development of many organs through integrin-mediated signaling events. Our results revealed that integrin β_1_ disruption resulted in decreased cardiomyocytes differentiation, but whether other cell lineages will be affected by integrin β_1_ disruption remained to be further investigated (Additional file 1: Figure S1). Moreover, the potential mechanism remained unclear, although we excluded the possibility that the loss of pluripotency is not affected by integrin β_1_ blocking (Additional file 2: Figure S2). These effects of integrin disruption may relate to the loosely formed EBs which lack some structure that are essential for cardiogenesis.

## Conclusions

In summary, our study demonstrated that blockage of collagen/β_1_ integrin interaction inhibited the quality and growth of miPSC-derived EBs, resulting in reduced EB size and decreased cardiac differentiation potential. These findings indicated the essential role of collagen/β_1_ integrin interaction in cardiac lineage commitment of miPSCs during EB formation, and will shed light on engineering matrix microenvironment within EBs to efficiently direct the cardiac fate of pluripotent stem cells.

## Methods

### Culture of mESCs and miPSCs

The miPSCs used in this study (kindly provided by Duanqing Pei, Chinese Academy of Sciences), which carry the GFP transgene targeted to the Oct4 locus, were generated by retroviral transduction of adult fibroblasts from OG2 mice with pMX-based retroviral vectors encoding the transcription factors Oct4 and Sox2. Undifferentiated mESCs (CGR8) and miPSCs were cultured on a mitotically inactivated mouse embryonic fibroblast (MEF; 50,000 cells/cm^2^) feeder layer or on gelatin-coated dishes as described previously [29]. The culture medium consisted of 85% knockout high-glucose glutamine-free DMEM with sodium pyruvate, supplemented with 15% serum replacement, 2 mM GlutaMAX, 0.1 mM nonessential amino acid stock, 0.1 mM β-mercaptoethanol (all from Invitrogen, Carlsbad, CA), and 1000 U/ml murine leukemia inhibitory factor (Chemicon, Temecula, CA). The culture medium was changed daily, and the cells were passaged every 2–3 days to maintain their undifferentiated state.

### EB formation via hanging drop method and static suspension culture

Before the initiation of differentiation, mESC and miPSC colonies were passaged up to 3 times on gelatin-coated dishes without feeder cells to eliminate contaminating MEFs. Before EB induction, adherent cells were enzymatically dissociated into single cells using 0.05% Trypsin-EDTA (Invitrogen). For the hanging drop method, EBs were formed in hanging drops consisting of 500 cells in 20 μl of differentiation medium. For static suspension culture, dissociated cells were cultured to form spheroids (EBs) using a 3-dimensional culture system in the differentiation medium on uncoated Petri dishes (Greiner Bio-One, Monroe, NC). At day 3 of differentiation, the EBs were transferred onto ultra-low attachment 6-well plates (Corning, Cat. No. 3471) in suspension culture for 2 additional days. The differentiation medium was based on high-glucose glutamine-free DMEM and supplemented with 20% ES-qualified fetal bovine serum (Hyclone, Logan, UT), 2 mM GlutaMAX, 0.1 mM nonessential amino acid stock, and 0.1 mM β-mercaptoethanol.

To elucidate the role of collagen/β_1_ integrin interaction in EB formation, ascorbic acid (Vc, collagen stimulator; 100 μM, Sigma) or cis-4-hydroxy-D-proline (CIS, collagen inhibitor; 300 μM, Sigma) was administered prior to EB formation. In some experiments, 10 μg/ml β_1_ integrin blocking antibody (clone Ha2/5, BD Pharmingen) was administered to block collagen/β_1_ integrin interaction.

### Morphological analysis of EBs

Gross morphology was analyzed to determine whether the blocking of collagen/β_1_ integrin interaction affected the growth of EBs from mESCs and miPSCs. Images of EBs formed by the hanging drop method and by static suspension culture under various conditions were obtained on days 3 and 5. For size analysis, mean diameters were measured using the Image J software program and calculated from more than five sample EBs per field in 10 fields as described previously [30]. When the significant differences were analyzed, the maximum and minimum of diameters in each group were removed to avoid large error. Three or more independent experiments were performed for each time point.

### Cardiac differentiation of EBs

Day 5 EBs were plated on 0.1% gelatin-coated 6-well culture plates at a density of ≈ 10 EBs per well. Starting at day 6 of differentiation, the differentiation medium described above was replaced every second day, and each EB outgrowth was examined daily for areas that displayed spontaneous beating.

### Immunocytochemistry

Whole EBs (day 3) and plated EBs (day 21) were stained to detect collagen and cardiomyocyte proteins, respectively. For Collagen I staining, whole iPSCs-derived EBs (day 3) were fixed in 4% paraformaldehyde for 40 min and embedded for frozen sectioning using OCT. Plated EBs (day 21) were fixed in 4% paraformaldehyde for 15 minutes at room temperature. All samples were permeabilized in 0.1% Triton X-100 for 10 minutes, and then blocked with 5% BSA for 30 minutes. The cells were incubated overnight with rabbit monoclonal anti-cardiac troponin T antibody (cTnT; Abcam, 1:100) and rabbit monoclonal anti-collagen type I antibody (Abcam, 1:100). After incubation overnight, we identified that there were no Oct4-GFP residue in samples using a fluorescence microscope before incubation with the FITC-conjugated secondary antibody. The bound antibodies were visualized by incubation with the appropriate secondary antibody (goat anti-rabbit IgG-FITC; Abcam, 1:500) for 40 minutes at 37°C. Then the cells were counterstained with DAPI (Sigma, 1:1000) and analyzed using a fluorescence microscope.

### Semiquantitative RT-PCR and quantitative real-time PCR

Total RNA was extracted from EBs using Trizol reagent (Invitrogen) according to the manufacturer’s instructions. Following treatment with Recombinant DNAse I (Takara, Japan) to remove genomic DNA contamination, 400 ng/μl RNA was reverse transcribed using the PrimeScript™ First Strand cDNA Synthesis Kit (Takara). The resulting cDNA was amplified by polymerase chain reaction (PCR) using standard methods. Primer sequences and PCR conditions are detailed in Additional file 3: Table S1 and Additional file 4: Table S2. mRNA levels of the target genes were normalized to β-actin or GAPDH. Three replicates were performed for each time point.

SYBR Green real-time PCR studies were performed using SYBR Premix Ex Taq™ II (Takara). All experiments were conducted in triplicate. Samples were subjected to the following cycle 40 times: 5 minutes at 95°C; 40 cycles of 30 seconds at 95°C, 30 seconds at 60°C, and 30 seconds at 72°C; a final step of 10 minutes at 72°C and 5 minutes at 4°C. The size of amplicons and the absence of nonspecific products were measured or confirmed using melting curves and gel electrophoresis. The transcripts for β-actin and GAPDH were used for internal normalization. Relative quantification was performed by the △△C_T_ method.

### Scanning electron microscopic analysis

mESCs and miPSC-derived EBs (days 3 and 5) were fixed for 2 h in 2.5% glutaraldehyde diluted in sodium cacodylate buffer (Electron Microscopy Sciences) and post-fixed with 1% osmium tetroxide buffered with 0.1 M sodium cacodylate for 1 h. The fixed samples were dehydrated using a series of ethanol solutions of increasing concentration (50%, 70%, 80%, 95%, 100%), critically point dried with CO_2_, and sputter-coated for 120 s at 2.0 KV using a Polaron SC7640 sputter coater. The samples were observed and photographed using a Philips XL20 scanning electron microscope.

### Statistical analysis

The results are presented as the mean ± SEM. The data were analyzed by Student’s t-test or 1-way ANOVA with repeated-measures analysis. For all analyses, differences with *p* values < 0.05 were considered statistically significant.

## Abbreviations

ESC: embryonic stem cell; iPSC: induced pluripotent stem cell; EB: embryoid body; ECM: extracellular matrix; Vc: ascorbic acid; CIS: cis-4-hydroxy-D-proline.

## Competing interests

The authors declare that they have no competing interests related to the manuscript.

## Authors’ contributions

QSZ conceived of the study, participated in its design and revised manuscript critically for important intellectual content. DZ and DBO performed most experiments in immunocytochemistry, scanning electron microscopic analysis, PCR, cell culture and cardiac differentiation of miPSCs. TW and LD analyzed the data. XL, XLH and XTL revised the paper. All authors read and approved the final manuscript.

## Supplementary Material

Additional file 1: Figure S1 Expression of markers related to three germ layer within EBs. Semiquantitative RT-PCR measurement of markers related to the three germ layer (endoderm: a-Fetoprotein, GATA-6; mesoderm: Brachyury; ectoderm: TuJ1, Map2) within 3d- and 5d-EBs derived from cells subject to integrin disruption and controls. Experiments were performed in triplicate, and the transcripts for GAPDH were used for internal normalization. Click here for file

Additional file 2: Figure S2 Loss of pluripotency within EBs after integrin disruption. Semiquantitative RT-PCR (A) and quantitative PCR (B, C) measurement of pluripotent markers (OCT3/4 and Nanog) within 3d- and 5d-EBs derived from cells subject to integrin disruption and controls. Expression levels of each gene were normalized to GAPDH. Mean fold change relative to GAPDH and SD from triplicate experiments are shown. Click here for file

Additional file 3: Table S1 Primers and cycling conditions for RT-PCR. Click here for file

Additional file 4: Table S2 Primers for RT-PCR in supplementary figures. Click here for file
