# Supplementary figures and images for: Collagen/β1 integrin interaction is required for embryoid body formation during cardiogenesis from murine induced pluripotent stem cells
Source: BMC Cell Biol. 2013 Jan 25;14:5. doi: 10.1186/1471-2121-14-5 (PMC3562267; doi:10.1186/1471-2121-14-5)

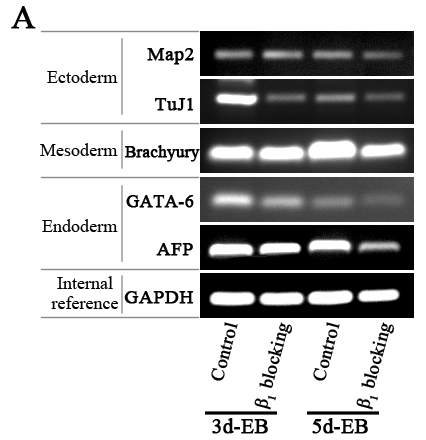

Supplement: Additional file 1: Figure S1 — Expression of markers related to three germ layer within EBs. Semiquantitative RT-PCR measurement of markers related to the three germ layer (endoderm: a-Fetoprotein, GATA-6; mesoderm: Brachyury; ectoderm: TuJ1, Map2) within 3d- and 5d-EBs derived from cells subject to integrin disruption and controls. Experiments were performed in triplicate, and the transcripts for GAPDH were used for internal normalization. [file 1471-2121-14-5-S1.tiff]

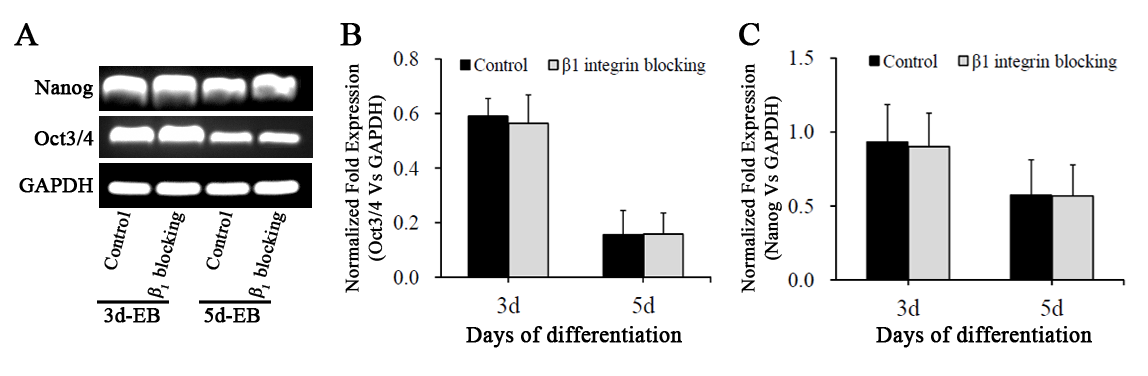

Supplement: Additional file 2: Figure S2 — Loss of pluripotency within EBs after integrin disruption. Semiquantitative RT-PCR (A) and quantitative PCR (B, C) measurement of pluripotent markers (OCT3/4 and Nanog) within 3d- and 5d-EBs derived from cells subject to integrin disruption and controls. Expression levels of each gene were normalized to GAPDH. Mean fold change relative to GAPDH and SD from triplicate experiments are shown. [file 1471-2121-14-5-S2.tiff]
