# Supplementary material for: Collagen/β1 integrin interaction is required for embryoid body formation during cardiogenesis from murine induced pluripotent stem cells
Source: BMC Cell Biol. 2013 Jan 25;14:5. doi: 10.1186/1471-2121-14-5 (PMC3562267; doi:10.1186/1471-2121-14-5)
Supplement: Additional file 3: Table S1 — Primers and cycling conditions for RT-PCR. [file 1471-2121-14-5-S3.doc]

**Table S1: Primers and cycling conditions for RT-PCR**

| Target Gene | GeneBank Number | Primer Sequence (5’-3’) | Size (bp) | Tm |
| --- | --- | --- | --- | --- |
| Col1A1 | *NM_007742* | CAAGGTCCTTCTGGATCAAGTG CCTTTATGCCTCTGTCACCTTG | 373 | 57 |
| Col1A2 | *NM_007743* | TGTTGGCCCATCTGGTAAAGA CAGGGAATCCGATGTTGCC | 113 | 60 |
| Col3A1 | *NM_009930* | GACCAAAAGGTGATGCTGGACAG CAAGACCTCGTGCTCCAGTTAG | 117 | 60 |
| Itga1 | NM_001033228 | TGGCCAACCCAAAGCAAGAA AGGGCCCACATGCCAGAAAT | 200 | 60 |
| Itga2 | NM_008396 | TGTGCACCCCCAGAGCACTT TGTTCACTTGAAGGCCCGGA | 181 | 60 |
| Itgb1 | NM_010578 | TCTCACCAAAGTAGAAAGCAGGGA ACGATAGCTTCATTGTTGCCATTC | 138 | 60 |
| GATA4 | *NM_008092* | TCAAACCAGAAAACGGAAGC GTGGCATTGCTGGAGTTACC | 117 | 60 |
| Mef2c | *NM_025282* | AGATACCCACAACACACCACGCGCC ATCCTTCAGAGAGTCGCATGCGCTT | 196 | 60 |
| Nkx2.5 | *NM_008700* | AAGTGCTCTCCTGCTTTCCCAG TTGTCCAGCTCCACTGCCTTC | 131 | 60 |
| a-MHC | *NM_010856* | TGAAAACGGAAAGACGGTGA TCCTTGAGGTTGTACAGCACA | 132 | 60 |
| MLC2a | *NM_022879* | TCAGCTGCATTGACCAGAAC AAGACGGTGAAGTTGATGGG | 148 | 60 |
| MLC2c | *NM_010861* | AAAGAGGCTCCAGGTCCAAT CCTCTCTGCTTGTGTGGTCA | 177 | 60 |
| β-Actin | GI 6671508 | CAAGAGATGGCCACTGCC CTTGATCTTCATGGTGCTAGGA | 312 | 55 |
| GAPDH | NM_008084 | TGTGTCCGTCGTGGATCTGA TTGCTGTTGAAGTCGCAGGAG | 150 | 60 |

**Abbreviations:** *GATA4*, GATA-binding protein 4; *Nkx2.5*, NK2 transcription factor related, locus 5; *Mef2c*, myocyte enhancer factor 2C; *AFP*, α-fetoprotein; *α-MHC*, α-myosin heavy chain; α-MHC, α-myosin heavy chain; *MLC2a,* myosin light chain 2 atrial transcripts; *MLC2v*, myosin light chain 2 ventricular transcripts; *GAPDH,* glyceraldehyde-3-phosphate dehydrogenase; *RT-PCR*, real-time PCR; *bp*, base pairs.
