# Supplementary material for: Collagen/β1 integrin interaction is required for embryoid body formation during cardiogenesis from murine induced pluripotent stem cells
Source: BMC Cell Biol. 2013 Jan 25;14:5. doi: 10.1186/1471-2121-14-5 (PMC3562267; doi:10.1186/1471-2121-14-5)
Supplement: Additional file 4: Table S2 — Primers for RT-PCR in supplementary figures. [file 1471-2121-14-5-S4.doc]

**Table S2: Primers for RT-PCR in supplementary figures**

| Target Gene | GeneBank Number | Primer Sequence (5’-3’) |
| --- | --- | --- |
| GATA6 | *NM_010258* | TTAACACTGATTGCTGCAACG GTTCATCGTAACGTGGCTGA |
| a-Fetoprotein | *NM_007423* | TCGTATTCCAACAGGAGG CACTCTTCCTTCTGGAGATG |
| Brachyury | *NM_009309* | CATCGGAACAGCTCTCCAACCTAT GTGGGCTGGCGTTATGACTCA |
| TuJ1 | NM*_011655* | TCACTGTGCCTGAACTTACC GGAACATAGCCGTAAACTGC |
| Map2 | *NM_0013066* | CATCGCCAGCCTCGGAACAAACAG TGCGCAAATGGAACTGGAGGCAAC |
| Oct3/4 | *NM_013633* | GCGTTCTCTTTGGAAAGGTG AGCCTCATACTCTTCTCGTTGG |
| Nanog | *NM_028016* | GGACAGGTTTCAGAAGCAGAAG ACCATTGCTAGTCTTCAACCAC |

**Abbreviations:** *GATA6*, GATA-binding protein 6; *TuJ1,* tubulin beta 5 class 1; *Map2,* microtubule-associated protein 2.
